# Investigation of mosquito oviposition pheromone as lethal lure for the control of *Aedes aegypti* (L.) (Diptera: Culicidae)

**DOI:** 10.1186/s13071-015-0639-2

**Published:** 2015-01-15

**Authors:** Song-Quan Ong, Zairi Jaal

**Affiliations:** Vector Control Research Unit, School of Biological Sciences, Universiti Sains Malaysia, 11800 Penang, Malaysia

**Keywords:** *Aedes aegypti*, Oviposition pheromone, Caproic acid, Temephos, Lethal lure

## Abstract

**Background:**

The trend in chemical insecticide development has focused on improving the efficacy against mosquitoes while reducing the environmental impact. Lethal lures apply an “attract-and-kill” strategy that draws the insect to the killing agent rather than bringing the killing agent to the insect.

**Methods:**

In this study, the mosquito oviposition pheromone was extracted from the eggs of *Aedes aegypti* (L.) and further investigated with a combination of pheromone and granular temephos as a lethal lure.

**Results:**

The compound caproic acid attracted significantly more egg-laying mosquitos at 1 ppm (660.83 ± 91.61) than the control (343.83 ± 56.24), which consisted of solvent only (Oviposition Activity Index: 0.316). Further investigation of the combination of caproic acid with granular temephos as a lethal lure attracted significantly more gravid female *Ae. aegypti* to oviposit their eggs than the temephos treated water and control.

**Conclusions:**

This indicated the ability of caproic acid in acting as an attractant and counters the repellency effect of temephos. Additionally, the presence of temephos in the lethal lure also restricted the hatching of the eggs and killed any larvae that hatched.

## Background

*Aedes aegypti* (L) is an important vector of mosquito-borne diseases that are frequently found in tropical and subtropical areas of the world [1]. The favorable climate and availability of vertebrate hosts in south-east Asia provide an excellent habitat that supports a high diversity of medically important mosquitoes. In Malaysia, *Ae. aegypti* is the most important species due to its role in the transmission of dengue viruses [2]. Insecticides have been the mainstay for mosquito control because they immediately suppress mosquito populations [2-4]. However, they have also caused undesirable effects such as the development of insecticide resistance, destruction of non-target organisms and endangerment to human health though exposure by handling and/or consumption of the insecticide [3,4] Furthermore, depending on the application method and climatic factors, only 10% of conventionally applied insecticides may reach their target in sufficient time and quantities [5].

The “attract-and-kill” approach was reviewed as an alternative solution in mosquito control [6]; this approach utilizes an attractant that draws the insect to the killing agent, as opposed to humans bringing the killing agent to the insect [7]. The strategy is target specific because the semiochemicals used are frequently species specific, maximizing mosquito-insecticide contact compared with other applications such as fogging and aerosols. Nonetheless, attract-and-kill studies have seldom been used on *Ae. aegypti* for oviposition or host-seeking because of the complex mixture of the attractants and the difficulty in determining the killing agent. Attempts at a lethal lure have been described by Okumu et al. [8], Smallegange and Takken [9], Ferdinand *et al*. [10] and Nancy *et al*. [11], who demonstrated that an odor-baited mosquito trap is effective, especially when integrated with other control methods. Nevertheless, the chemical ecology of mosquito semiochemicals is still poorly understood, and more studies are required to develop a better approach to mosquito control [12]. In this study, attractants are extracted using modified AOAC fatty acid extraction and identified using HPLC. The compounds' attractiveness was confirmed by conducting an attractiveness bioassay, and the attractant (OAI > 0.30) will become the candidate for the temephos lethal lure combination at the end of the experiment.

## Methods

### Insects and eggs

The eggs and adult females of the susceptible strain WHO/VCRU of *Ae. aegypti* were obtained from the Vector Control Research Unit (VCRU), University Science Malaysia. The mosquitoes were cultured at 27 ± 1°C and 75 ± 5% relative humidity in insectariums. The larvae were reared in dechlorinated water and fed with lab food (Dog biscuit: yeast: milk powder: beef liver powder at a 3:1:1:1 ratio). The pupae were transferred into a 30 × 30 × 30 cm netted cage for adult emergence. The adult mosquitoes were fed with 10% sucrose mixed with a Vitamin B complex as an energy supply. Fresh eggs (laid less than 24 hours) were used in the extraction, and the bioassay laboratory tests were performed on 4- to 5-day-old gravid *Ae. aegypti* females.

### Extraction of the mosquito oviposition pheromone (MOP)

The extraction method was slightly modified from the AOAC 969.33 fatty acid extraction method [13]. *Ae. aegypti* eggs were first preserved as lyophilized cells (freeze-dried cells) using a freeze-dryer and then converted into a non-reactive form by acid-catalyzed esterification [14]. The glycerol moiety of the lipids was replaced by methanol, and a non-reactive methyl ester was formed.

For the extraction of fatty acids, approximately 50 000 eggs of *Ae. aegypti* were immersed in 10 ml of methanol and frozen in liquid nitrogen (-196°C). Next, the sample was sent to the Biochemical Laboratory, School of Biological Sciences, University Sains Malaysia, to be freeze-dried using the Labcanio-Model 117 (England) freeze-dryer. The freeze-dryer kept the sample at -47°C with continuous pumping using a vacuum pump. After 48 hours, the samples became lyophilized cells.

The preparation for HPLC analysis was modified from the Folch procedure, which is an acid-catalyzed esterification. The freeze-dried cells were divided into 0.10- g samples using an electric microbalance and transferred vinto a small test tube. A total of five replicates were prepared. Two milliliters of solvent (85% sulfuric acid + 15% methanol) followed by 2 ml of chloroform were added. After adding the solvent, nitrogen was introduced to displace the air inside the test tube. The samples were placed in a heater and heated to 100°C for 30 minutes. Later, the samples were cooled down and then vortexed for two minutes. The samples were allowed to settle down overnight, enabling the mixture to form two layers of liquids. The bottom layer was removed and kept in a small Scott bottle for High Performance Liquid Chromatography (HPLC) analysis in the Biochemistry Laboratory of the School of Biological Sciences, University Science Malaysia.

HPLC (Perkin Elmer) used a two-solvent delivery pump, a U6K injector and a fatty acid silica-fused capillary column (Omegawax by Supelco, United States; dimensions: 30 meters in length, 0.25 mm in diameter and 0.25 μm in thickness). The flame-ionization detector was connected to the computer and a Model 990 Plotter. The HPLC was run using an isocratic gradient with a mobile phase composed of tetrahydrofuran–acetonitrile-water-acetic acid. The effluents were monitored over a range of 190-240 nm. The samples were injected into the column, and an internal standard was used to confirm the fatty acid.

### Chemicals

The tested HPLC-grade chemicals were purchased from Sigma-Aldrich Malaysia. Stock solutions were prepared from the raw chemicals, and hexane was used as the solvent. Each stock solution underwent a series of dilutions to obtain 1, 10 and 100 ppm concentrations. However, the 10% granular temephos, Abate® 1.1G (1.1% w/w; BASF, Malayisa), was used in the lethal lure part of the experiment.

### Attractiveness bioassay

A laboratory bioassay of oviposition attractiveness was performed in net cages measuring 30 × 30 × 30 cm with an opening on one side. A 90-mm diameter Whatman No. 1 filter paper was shaped into a cone, and the pointed end was cut off. The filter paper was then immersed in a paper cup, measuring 3 cm in height and 5 cm in diameter, containing 19 ml of dechlorinated water with 1 ml of solvent or 1 ml of MOPs at the desired concentration (1, 10 or 100 ppm).

Twenty 4- to 5-day-old gravid mosquitoes were released into the net cage, and the treated paper cups and the controls were subjected to the bioassay for 22 hours. The four paper cups (control, 1, 10 and 100 ppm treatments) were placed equidistant from each other. The paper cups were removed every day and replaced with fresh sample solutions. The paper cups were rotated clockwise with every change of fresh sample solution to prevent site selection by the mosquitoes. There were six replicates for each assay per day. Each chemical was evaluated separately. Experiments were conducted for 5 continuous days, and egg counts were conducted every day using a needle and a magnifying glass.

The measurement of the oviposition response was the number of eggs in both the control and treatment dishes. The oviposition activity was expressed as the oviposition activity index (OAI) by Kramer & Mulla [15]:

$$ \mathrm{O}\mathrm{A}\mathrm{I}={\mathrm{N}}_{\mathrm{T}}\hbox{--}\ {\mathrm{N}}_{\mathrm{S}}/\ {\mathrm{N}}_{\mathrm{T}}+{\mathrm{N}}_{\mathrm{S}} $$

where N_T_ denotes the mean number of eggs in the treated water and N_S_ denotes the mean number of eggs laid in the control water. All index values were within the range of +1 to −1. As suggested by Kramer and Mulla [15], compounds with an OAI of +0.30 or greater are considered attractants, whereas those with an OAI of −0.30 or less are considered repellents.

### Lethal lure bioassay

A laboratory bioassay was conducted in a 30 × 30 × 30 cm netted cage with an opening on one side. Twenty 4- to 5-day-old female *Ae. aegypti* were transferred to the experimental cages. The gravid female mosquitoes were allowed to select the oviposition site among the treatment oviposition cups containing the desired concentration of the attractant compound and granular temephos, the cups containing granular temephos in dechlorinated water and the control cups. They were placed in a triangular position with equal distance. Ten replicates were performed for each sample. The bioassay was performed for 22 hours, after which the old treatment and control cups were replaced with new ones. The bioassay was conducted for 5 continuous days. The positions of the paper cups were changed alternately to avoid any site selection.

The *Ae. aegypti* eggs were counted once they were collected. The counting was accomplished using a magnifying glass, a compound microscope and a needle. Eventually, the counted eggs were transferred unconditionally to an enamel tray (diameter 25 cm; height 4 cm) containing 1 l of dechlorinated water. Each tray contained 200 larvae to avoid overcrowding. The number of 1^st^ instar larvae was counted and considered to represent the hatchability; the mortality of the larvae after 24 hours was also studied.

### Statistical analysis

The attractiveness of the compound was prior analyzed based on the OAI, in which OAI values exceeding +0.30 were considered attractants, and confirmed by subjecting the eggs quantity of the attractant and control in SPSS 17.0 student t-test to compare their differences.

The effectiveness of the lethal lure was compared with the control. Three factors were studied: attractiveness (measurement of the number of eggs), egg hatching and mortality of the larvae. The results of the bioassay were subjected to One-way ANOVA by using SPSS 17.0.

## Results

### Identification of mosquito oviposition pheromone

**Figure 1:**
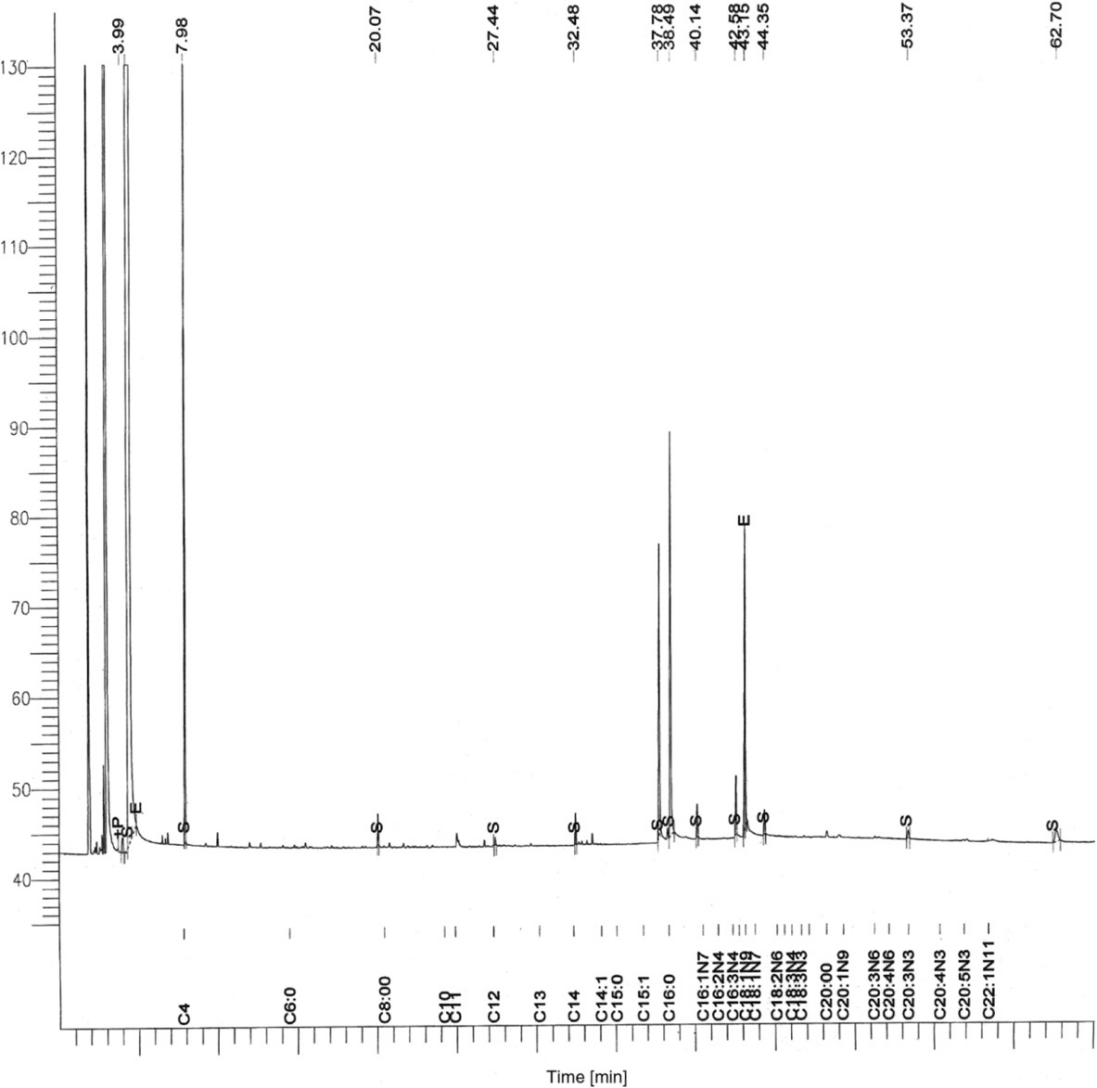
**Chromatograph of fatty acid extraction from** ***Aedes aegypti*** **mosquito eggs.**

The fatty acid extraction was identified according to Holman *et al*. [16] omega nomenclature or n nomenclature. The “n” indicates the number of carbons; the “x” indicates the double bond located on the x^th^ carbon–carbon bond, counting from the terminal methyl carbon (designated as n or ω) towards the carbonyl carbon. All the compounds were named according to SGE Analytical Science [17] and the Lipid library from Lipomics Technologies [18] (Table 1), and the respective peaks of the compounds are shown in the chromatograph in Figure 1.

**Table 1:** **Compounds extracted by fatty acid extraction and identified by HPLC**

**No**.	**Retention time (minute)**	**Compound name**	**Area (%)**
1.	4.344	C4:0 Butyric acid	93.86
2.	7.975	C6:0 Caproic acid	1.89
3.	20.073	C10 Capric acid	0.08
4.	27.436	C13 Tridecanoic acid	0.02
5.	32.480	C14:1 Myristoleic acid	0.08
6.	37.775	C16:0 Palmitic acid	0.92
7.	38.486	C16:1n7 Palmitoleic acid	1.45
8.	42.582	C18:1n9 Elaidic (t) or Oleic (c)	0.17
9.	43.147	C18:1n7 vaccenic acid	0.64
10.	43.155	C18:2n6 Linolelaidic (t) or linoleic (c)	0.42
11.	44.353	C18:3n6 g-Linolenic	0.08
12.	53.370	C20:4n3 eicsoatetraenoic acid	0.04
13.	62.699	C22:5n3 docosapentaenoic acid (DPA)	0.12
14.	65.616	C22:6n3 docosahexaenoic acid (DHA)	0.06

HPLC. high performance liquid chromatography.

### Oviposition response of gravid females of *Ae. aegypti* to the MOP

Due to the complexity of the mixture, only selective compounds were tested in this study. The selection is based on short-chain fatty acids that have fewer than 16 carbons [19]. Therefore, based on the results of the extraction of MOPs, the compounds tested were C6 caproic acid, C16 palmitic acid and C16:1n7 palmitoleic acid.

Hwang *et al*. [20] tested C4:0 butyric, C10 capric acid, C13 tridecanoic acid and C14:1 myristoleic acid on *Ae. aegypti*, but these compounds were not tested in the present study.

Caproic acid is a saturated six-carbon fatty acid. The fatty acid attracted significantly more mosquitoes compared with the control at 1 ppm. The OAI for the 1 ppm caproic acid was 0.316 and the number of eggs laying induced by caproic acid (660.83 ± 91.61) is significantly higher than the control (343.83 ± 56.24) at p < 0.05. Nevertheless, when caproic acid was tested at the higher concentrations of 10 ppm and 100 ppm, the results were still positive but were not considered as an attractant. Palmitic acid is a saturated fatty acid that contains 16 carbons. The compound attracted fewer mosquitoes than the control at all concentrations but was not considered a repellent based on its OAI value. In contrast, palmitoleic acid showed negative attractiveness towards *Ae. aegypti* at all concentrations. The mean number of eggs and the OAI values are shown in Table 2.

**Table 2:** **Oviposition responses of the gravid females** ***Aedes aegypti*** **to the MOP**

**MOP**	**Concentration**	Mean number of eggs**	**OAI**
Caproic acid (Hexanoic acid)	Control	343.83 ± 56.24	
	1 ppm	660.83 ± 91.61*	0.316
	10 ppm	345.00 ± 54.89	0.002
	100 ppm	411.17 ± 114.88	0.089
Palmitic acid (Hexadecanoic acid)	Control	560.67 ± 60.39	
	1 ppm	449.00 ± 69.24	-0.111
	10 ppm	536.33 ± 96.49	-0.022
	100 ppm	495.00 ± 70.00	-0.062
Palmitoleic acid (9-hexedecanoic acid	Control	510.82 ± 140.89	
	1 ppm	397.00 ± 106.20	-0.125
	10 ppm	413.83 ± 121.07	-0.105
	100 ppm	317.83 ± 87.36	-0.233

OAI. oviposition activity index.

t (18), *P < 0.05.

**Statistical test was only carried out on the attractant which OAI exceed 0.3.

### Oviposition responses of *Ae. aegypti* to a combination of lethal lure

The lethal lure, which consisted of temephos and caproic acid, attracted a significantly higher number of eggs (835 ± 84) compared with the control (F: 1.08; df: 18; P < 0.001). The control, which consisted of only hexane (solvent), contained 538 ± 74 eggs laid by the gravid *Ae. aegypti*. On the other hand, temephos has attracted a significantly lower number of eggs (311 ± 64) than the lethal lure and control, in which indicating its repellency properties. Furthermore, the lethal lure and temephos showed relatively low egg hatch (3% and 4%, respectively of the total number of eggs) and good mortality (92% and 95%, respectively) compared with the control, which showed a higher percentage of egg hatch (87% of the total number of eggs) (Table 3).

**Table 3:** **Oviposition responses of Aedes aegypti to the control (Hexane) and treatment (Caproic Acid and Temephos) and the percentage of post-treatment hatching and mortality**

**Sample**	**Mean ± S.E.**	**OAI**	Hatching (%)*	Mortality (%)**
Control (Hexane in dechlorinated water)	538 ± 75		87%	0%
Temephos (temephos and hexane in dechlorinated water	311 ± 64	-0.27	4%	95%
Lethal lure (caproic acid and temephos)	835 ± 94***	0.22	3%	92%

t (18), ***P < 0.001.

S.E. standard error.

OAI. oviposition activity index.

*a Percentage of hatching was based on total egg of each sample (control. 5383; temephos 5242; lethal lure 8354).

**b Percentage of mortality was based on the larvae hatch (control. 4523; temephos 209; lethal lure 212).

## Discussion

### Identification of mosquito oviposition pheromone

Although most of the extraction methods involved methanol being rinsed off by distilled water, a major disadvantage is that two distinct observable layers were not formed because methanol is completely miscible with water. Thus, the mixture of methanol and water will produce a homogeneous solution, and the MOP compounds would eventually be randomly distributed between the methanol and water layers. As a result, the MOP compounds are difficult to obtain by mixing methanol and water.

There were 14 fatty acids detected by the GC, and the results consisted of several compounds in high proportions. Butyric acid was the compound with the highest proportion in the analysis, similar to the extraction of the active fraction of 1% Lab Chow Infusion by Hwang *et al*. [20]. Caproic acid was also present in the 1% Lab Chow Infusion. These findings could indicate that butyric acid and caproic acid originated from the eggs of the mosquito and not from organic debris produced via the fermentation of bacteria in the infusion. Moreover, palmitic acid and palmitoleic acid were also present in the egg extraction conducted by Ganesan *et al*. [6]. However, the palmitoleic acid detected by HPLC was not specified in the geometric isomerism compared with the attractive (Z)-9-hexadecanoic acid tested by Ganesan *et al*. [6]. The fatty acid extraction improved the identification of shorter fatty acid chains, suggesting that freeze-drying the eggs can eventually preserve some of the MOPs in the eggs.

### Oviposition response of gravid females of *Ae. aegypti* to the MOP

Freshly blood-fed female *Ae. aegypti* rested on the walls of the net cage. Approximately 24 hours were required for the blood to be completely digested and for eggs to form. The mosquitoes began laying their eggs on the second day after blood feeding. Female do not normally lay their entire batch of eggs in one location, but rather they distribute them in multiple water-filled containers, a behavior called “skip oviposition” [21]. However, the augmentation of some containers with organic materials can counteract skip oviposition and significantly increase the number of eggs in target containers [22]. Therefore, to minimize the skip oviposition behavior of the mosquitoes, MOPs were replaced every 22 hours to ensure a fresh effect.

The cues for the oviposition of mosquitoes depend on two factors: chemical and tactile [14]. Apart from the chemicals tested, cone-shaped filter paper was provided as the tactile stimulus, serving as an object for the mosquitoes to hold onto while ovipositing their eggs. Moreover, the presence of the filter paper prevented the mosquitoes from drowning while they were ovipositing their eggs. The number of eggs may differ from one cage to another according to the amount of blood ingested during a blood meal.

According to Kramer and Mulla [15] an OAI value of more than 0.300 indicates that the compound is an attractant. Caproic acid was one of the fatty acids tested by Kramer *et al*. [23] on the oviposition of *Culex quinquefasciatus*. However, caproic acid had not been previously tested on *Ae. aegypti*. In the present study, 1 ppm caproic acid attracted more gravid female mosquitoes to lay eggs than the control. In addition, the OAI value of 1 ppm caproic acid was +0.316, indicating that it is considered an attractant based on the definition by Kramer *et al*. [23]. Nevertheless, caproic acid repelled *Culex quinquefasciatus* Kramer *et al*. [23] but attracted *Ae. aegypti*, suggesting that mosquitoes reduced inter-species competition at the breeding site that could compromise survivorship between them.

Ganesan *et al*. [6] tested palmitic acid against *Ae. aegypti* at 1, 10 and 100 ppm concentrations, and the mosquitoes showed slight attraction at 1 and 10 ppm but were repelled at 100 ppm. This finding is in contrast with that of the present study, in which palmitic acid repelled mosquitoes at all concentrations. The OAI values for 1, 10 and 100 ppm palmitic acid were 0.111, -0.021 and -0.062, respectively.

However, racemic palmitoleic acid showed negative attractiveness at 1, 10 and 100 ppm, with OAI values of -0.125, -0.105 and -0.233, respectively. Nonetheless, Ganesan *et al*. [6] showed that the isomer (Z)-9-hexadecanoic acid (palmitoleic acid) attracted *Ae. aegypti* at concentrations of 10 and 100 ppm, with both showing OAI values of +0.55. The difference in the attraction response was due to the presence of the other isomer, (E)-hexadecanoic acid, in the racemic 9-hexadecanoic acid, which repelled *Ae. aegypti*. This result suggests that the (E)-isomer exhibited a repellent property, and this phenomenon also supports the high specificity of the compound to the olfaction of mosquitoes. As demonstrated by Ponnusamy *et al*. [14] microbial metabolites, which consist of fatty acids from carbon 16 to carbon 19, were implicated as mosquito attractants, but these odorants did not induce egg laying. This phenomenon was also observed in the present study, in which both hexadecanoic acid and racemic 9-hexadecanoic acid contained 16 carbons.

### Oviposition responses of *Ae. aegypti* to a combination of lethal lure

The lethal lure had a significantly higher number of eggs (835 ± 94, P < 0.001) laid in it than the control (538 ± 75). The current study on the combination of caproic acid and granular temephos as a lethal lure is reported for the first time in Malaysia. Previously, Nazni [24] attempted a combination of a field ovitrap and temephos against *Aedes* mosquitoes and found that the oviposition activity of *Aedes* mosquitoes was not significantly different from the field ovitrap alone. The result of the study by Nazni (2009) [24] supported the present study, in which temephos was not affecting the attractiveness of lethal lure. Although Humberto *et al*. [25] had demonstrated temephos post repellency properties when compared to another insecticide, spinosad, but the present study had showed the capability of caproic acid to reverse the repellency effect of temephos. This is demonstrated in the OAI of this study that lethal lure which contained caproic acid and temephos was 0.22 (attracting) while temephos that contained temephos in dechlorinated water was -0.26 (repelling). Michaelakis *et al*. [7] studied the combination of the synthetic oviposition pheromone 6-acetoxy-5-hexadecanolide and granulated temephos against *Culex pipiens*. The 300 mg of 6-acetoxy-5-hexadecanolide managed to attract the mosquitoes to lay a significant number of egg rafts on the treatment, and the temephos eventually killed the larvae that hatched. The results of Michaelakis *et al*. [7] in an attract-and-kill strategy using temephos are similar to the present study. Both studies indicated that the use of temephos did not affect the effectiveness of the attractant.

Temephos causes minimum undesirable effects on the environment and public health. Additionally, due to its relatively low mammalian toxicity, it is used as a larvicide in many mosquito control programs in various formulations [7,26,27].

The presence of temephos restricts the hatching of *Ae. aegypti* eggs, which is evident from the differences between the egg-hatching percentages of the control trap and the lethal trap; the control had a higher percentage than the lethal trap. Nevertheless, some of the larvae managed to hatch but were eventually killed by the temephos.

The attract-and-kill strategy is still rarely used for mosquito control in Malaysia; it is already used against insecticide-resistant Lepidoptera and is suggested as a new low-emission way to control insect pests [7,28]. Although the results were encouraging, further studies in the field are still required to determine the effect of the environment’s abiotic and biotic factors on the lethal lure. The addition of the attractant may increase the cost of mosquito control; however, the increase in cost is compensated for by the lower quantity of the insecticide used due to the more accurate insecticide-mosquito contact percentage in the lethal lure than the insecticide alone. Therefore, the attract-and-kill strategy, or lethal lure, is one potential control method that should be integrated with other control approaches for mosquito control.

## Conclusion

A caproic acid-temephos combination performed well in the laboratory as a lethal lure; caproic acid attracted more gravid mosquitoes, while temephos acted as the killing agent by restricting egg hatching and killing the larvae that hatched. From previous studies, developing an effective lethal lure consists of complex mechanisms and high manufacturing costs. However, in this experiment, the lethal lure used low cost caproic acid and temephos. In addition, the application mode of this lethal lure was not complicated: the temephos granules were added to a 1 ppm caproic acid solution. Further studies should focus on the feasibility of field application and the development of the lethal lure against the adult stage of *Ae. aegypti*.
